# Second Metacarpal Index as a Predictor of Secondary Displacement in Conservatively Treated Distal Radius Fractures

**DOI:** 10.3390/medicina62010105

**Published:** 2026-01-02

**Authors:** Alexandru Jecan, Gheorghe Tomoaia, Răzvan Marian Melinte, Diana Jecan-Toader, Roxana Cristina Rad Bodan, Daniel Oltean-Dan

**Affiliations:** 1 Department of Orthopedics and Traumatology, University of Medicine and Pharmacy “Iuliu Hatieganu”, 400132 Cluj-Napoca, Romania; jecan.alex@gmail.com (A.J.); olteandandaniel@yahoo.com (D.O.-D.); 2 Orthopedics and Traumatology Clinic “Alexandru Radulescu”, Emergency County Hospital, 400347 Cluj-Napoca, Romania; 3 Leon Daniello Pneumology Hospital, 400371 Cluj-Napoca, Romania; 4 2nd Pediatric Department, University of Medicine and Pharmacy “Iuliu Hatiganu”, 400132 Cluj-Napoca, Romania; 5 2nd Pediatric Clinic, Emergency Clinical Hospital for Children, 400124 Cluj-Napoca, Romania; 6 Faculty of Medicine, University “Dimitrie Cantemir” Târgu-Mureș, No. 3-5 Bodoni Sandor, 540545 Târgu-Mureș, Romania; roxibodan@gmail.com

**Keywords:** second metacarpal index, distal radius fracture, secondary displacement, osteoporosis

## Abstract

*Background and Objectives:* Distal radius fractures (DRFs) represent the most common fracture in the elderly population and are typically the first fractures to occur in the sequence of fragility fractures. Although fracture instability is an important criteria for guiding treatment, there is no universal consensus on how to define an unstable DRF. Given the demonstrated influence of bone quality on fracture stability, it has been hypothesized that second metacarpal index (2MCI) may also serve as a predictor of instability in DRFs. This study aimed to evaluate the predictive value of 2MCI for fracture instability and to determine its threshold value beyond which surgical management should be considered. *Materials and Methods:* A retrospective study was conducted between January 2023 and May 2025 investigating conservatively treated DRFs. Radiographic parameters including 2MCI, volar inclination, radial inclination, and ulnar variance were obtained at three time points: pre-reduction, post-reduction, and at 6 weeks of follow-up time. Univariate and multivariate linear regression analysis and receiver operating characteristic (ROC) analysis were conducted to determine the optimal 2MCI threshold for predicting clinically significant displacement. *Results*: A strong correlation was found between 2MCI and the changes (∆) in volar inclination (*p* < 0.001), radial inclination (*p* < 0.001), and ulnar variance (*p* < 0.001) in univariate regression analysis. In multivariate regression analysis, 2MCI was an independent predictor of ∆ volar inclination (*p* < 0.001) and ∆ radial inclination (*p* = 0.004). For ∆ ulnar variance, both 2MCI (*p* = 0.003) and initial displacement (*p* = 0.049) were statistically significant predictors. A 2MCI cut-off value of 0.412 predicted a ∆ volar inclination greater than 10° (sensitivity 80.9% and specificity 74.1%, *p* < 0.001). *Conclusions*: The results of this study reveal the potential of the 2MCI as a quantitative marker of both fracture instability and bone fragility. Further prospective validation may demonstrate its role as a standard radiographic parameter in DRFs, as well as in guiding treatment selection.

## 1. Introduction

Distal radius fractures (DRFs) are among the most common fractures, particularly in patients over 65 years, where they account for up to 18% of all fractures [1]. Historically, these injuries were managed by splinting [2]. With advances in modern medicine, various methods (volar plating, intramedullary nail and k wire fixation) of fracture osteosynthesis have been developed, offering improved outcomes in terms of bone consolidation, early mobilization, and fracture stability. Nevertheless, despite the widespread use of fixation devices such as volar plating, results remain controversial. Recent meta-analyses have shown only modest improvements in upper extremity function and grip strength in surgically treated patients while reporting worse outcomes regarding pain, surgical complications, and cost [3]. These results raise the question not of their utility but rather of the appropriate indication for surgical versus conservative treatment. A major limitation of conservative management lies in the difficulty of reliably identifying when a fracture is likely to become unstable.

Unstable DRFs and subsequent secondary displacement are the main cause of poor outcomes following conservative or surgical treatment [4]. Current tools and criteria used to assess DRF instability are highly subjective and observer-dependent, which limits their predictive accuracy for both treatment selection and clinical outcomes. At present, there are no universally accepted objective parameters capable of quantifying the risk of secondary displacement. This lack of standardization contributes to variability in clinical decision making and significant disagreement among physicians when determining the optimal treatment approach. Despite the high incidence of this injury and high number of studies on this subject, there is still no consensus on the definition of an unstable DRF. A recent systematic review reported that the most commonly used definition for an unstable fracture is the loss of position after adequate reduction or the Lafountaine criteria from 1989 [5]. While these criteria are still in use and provide important guidance in treatment selection, they primarily describe characteristics of the fracture anatomy. Age is the only factor that accounts for bone health, being associated with an increased incidence of osteoporosis, reported at 35.3% in elderly men and women around the world in a recent meta-analysis [6]. However, although age is an important predictor of osteoporosis risk at the population level, it is not a reliable tool for quantifying individual diseases severity; therefore, the degree of osteoporosis should be assessed by bone density scans (DEXA) or other validated measures [7].

Osteoporosis is an important predictor of bone healing and stability in both surgically and conservatively treated patients [8]. In the acute trauma setting, information on a patient’s osteoporotic status is rarely available, and a bone density scan (DEXA) is often not feasible. The second metacarpal index (2MCI), defined as the ratio of the total cortical thickness to the total width of the second metacarpal bone at its narrowest point, has been shown to be a reliable indicator of osteoporosis, with good correlation with DEXA measurements [9].

Several radiographic parameters have recently been proposed to describe and predict the evolution of DRFs. In 2024, Kitidumrongsook et al. introduced the ulnar axis as an alternative reference to the radial axis for taking the standard measurements of a DRF (radial inclination, volar inclination, ulnar variance), particularly useful when the radial axis is distorted, while Pace et al. proposed the Pacetti line as a novel radiological landmark to predict the secondary displacement of DRFs immediately after reduction and during early follow-up (at 7 and 14 days), reporting promising preliminary results [10,11]. While these methods offer interesting perspectives for improving radiographic assessment, they are not yet widely used in clinical practice, and further studies are needed to confirm the results and their clinical applicability in predicting fracture instability.

This study aims to assess the ability of the second metacarpal index to predict secondary displacement in conservatively treated distal radius fractures and to identify a threshold value at which conversion to surgical treatment should be considered.

## 2. Materials and Methods

### 2.1. Study Design, Patients, and Data Collection

We conducted a single-center retrospective cohort study of all patients treated in a tertiary trauma center between January 2023 and May 2025 for a DRF who were managed conservatively. Patients were identified by running a search in the electronic database of the hospital using International Classification Diagnosis codes for distal radius fractures. All consecutive patients with a distal radius fracture, at least 18 years old, who underwent conservative treatment in concordance with our internal guidelines for DRFs (described below), with pre- and post-reduction distal radius X-rays and a minimum of 6 weeks of radiological follow-up were included. The exclusion criteria were surgically treated patients, non-compliance with treatment, incomplete follow-up, poor-quality of X-rays, open fractures, pathological fractures, and previous ipsilateral wrist surgery or fracture.

A total of 295 patients were identified. Of these, 190 were excluded due to poor follow-up, non-compliance with treatment, or non-conservative treatment. The remaining 105 patients were included.

Guidelines of American Academy of Orthopedic Surgeons (AAOS) recommend considering a surgical treatment for fractures that present a displacement of >10° of dorsal angulation, >3 radial shortening, and intra-articular displacement > 2 mm [12]. According to the literature, a secondary displacement with functional implication is considered with a ∆ < 10° of volar inclination, <5° of radial inclination, and <2 mm of ulnar variance from the initial post-reduction radiographic measurements [13,14].

This study followed the principles outlined in the Declaration of Helsinki. Ethical approval was obtained from the local ethics committee prior to data collection (no.DEP243/23 July 2025 and 26646/23 July 2025). The requirement for informed consent was waived due to the retrospective design and the use of anonymized data.

### 2.2. Treatment Protocol

Our internal guidelines recommend the use of the following protocol: patients are treated with closed reduction under analgo-sedation or loco-regional anesthesia. Reduction is performed via one assistant applying manual traction while another provides counter-traction at elbow. The operator then manipulates the fragments into the correct position. Following reduction, temporary immobilization is carried out with an antebrachio-palmar cast, and a control radiograph is obtained. If the reduction is satisfactory, the cast is converted to a brachio-palmar half-pipe cast. Immobilization is maintained for 2 weeks above the elbow, followed by 4 weeks below the elbow. Clinical and radiographic follow-up are performed at 1, 3, 6, and 12 weeks. Although a six-week protocol appears strict and overly cautious, current guidelines for reduced DRFs recommend an immobilization of at least four weeks, extending up to six weeks, with no proven superiority or inferiority between the two durations and functional outcomes being comparable. Above-elbow casting is used during the period of highest fracture instability (first two weeks) and as a strategy to control rotational forces [15,16].

### 2.3. Data Collection

Demographic and radiological data were retrieved from our electronic database and anonymized prior to analysis. We collected demographic data about age and gender.

Radiological assessment included two orthogonal views (anteroposterior and lateral) pre- and post-reduction and at 6 weeks of follow-up. All radiographs were independently reviewed by one senior orthopedic surgeon (D.D.O) and one senior orthopedic surgery resident (A.J). Measurements were performed by both investigators, and any discrepancy greater than >1 mm or >0.5° were reviewed by a third senior orthopedic surgeon (R.M.M) with a consensus decision reached among the three investigators. The investigators were blinded to the 6-week outcomes when measuring the 2MCI. All the fractures were classified according to the AO distal radius classification [17]. The Lafontaine criteria were noted pre-reduction, and the following measurements were assessed: 2MCI, radial inclination, volar inclination, ulnar variance, and initial displacement.

**Figure 1:**
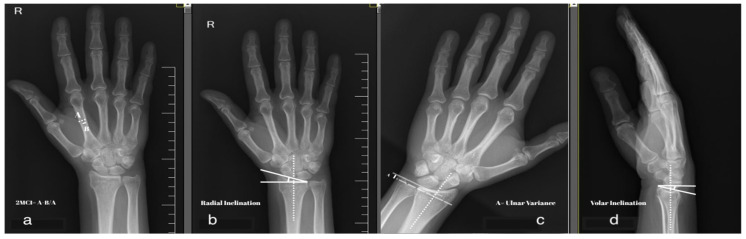
(**a**–**d**): Radiographic measurements.

The second metacarpal index was measured as the ratio between the total cortical width and the width of the second metacarpal at its narrowest point (middle) on an anteroposterior X-ray (Figure 1a). Radial inclination was measured on an AP view as the angle between the line drawn between the radial styloid and ulnar border of distal radius and a perpendicular line on the long axis of the radius (Figure 1b). Radial inclination was measured as the angle between a line that passes through the volar and dorsal rim of distal radius and a line perpendicular on radius long axis (Figure 1c). Ulnar variation was measured as the distance between the radius articular surface and the ulna (Figure 1d). Initial displacement was considered when the initial dorsal tilt of the fracture was >20°.

The collected data was used to calculate changes (∆) in radial inclination, volar inclination, and ulnar variance. These ∆ values were defined as the absolute difference between the immediate post-reduction measured value and the value recorded at 6 weeks.

### 2.4. Statistical Analysis

The statistical analysis was performed using IBM SPSS (Version 26, release 26.0.0.0 64 bit edition) (IBM Corp, Armonk, NY, USA) and Microsoft Excel [16.101.2 (25092825)] (Microsoft Corp., Redmond, WA, USA). The normality of continuous variables was assessed using the Shapiro–Wilk test. Data were described using proportions for categorical variables and means with standard deviations (SDs) for continuous normally distributed variables, respectively, with medians with interquartile ranges (IQRs) for non-normally distributed data. To evaluate potential associations between variables and outcomes, univariate analyses were initially conducted. Univariate linear regression was used for continuous predictors to identify potential continuous modifiers, and *t*-tests or Mann–Whitney U tests were applied for comparisons of normally or abnormally distributed data, respectively.

Variables found to be statistically significant in univariate analyses, as well as known potential confounders (sex), were included in multivariate linear regression models. Model assumptions were verified using Durbin–Watson for independence of errors, residual plots for linearity and homoscedasticity, and P-P plots for normality. Multicollinearity was assessed using correlation matrices and variance inflation factors. Outliers and influential points were evaluated using leverage values and Cook’s distance. A sensitivity analysis was performed after excluding significant outliers yielding results consistent with primary analysis.

A receiver operating characteristic (ROC) analysis was conducted to evaluate the ability of the statistically significant covariate identified in the multivariate model to predict a volar tilt change greater than 10°, a threshold considered clinically significant for functional outcomes. The area under the curve (AUC) was calculated to assess discriminative ability, and the optimal cutoff value for the 2MCI was determined using the Youden J statistic, maximizing sensitivity and specificity.

## 3. Results

### 3.1. Demographic and Clinical Description of the Patients Included

A total of 105 patients with distal radius fractures treated conservatively between January 2023 and May 2025, with a minimum follow-up of 6 weeks, were included in the study. There were 82 (78.1%) women and 23 (21.9%) men, with a median age of 68 years (*IQR*: 19, range 20–90). The majority of fractures were classified as AO type A3 (55.2%), followed by A2 (20%) and C1 (5.7%). Dorsal comminution was present in 69.5% of cases and intra-articular extension in 22.9%. Associated ulnar fractures were identified in 50.5% of patients, most commonly involving the base of the styloid process. Descriptive data are presented in Table 1.

The mean radial inclination before reduction was 16.65° ± 6.31°. The median radial inclination after reduction was 21.00° (IQR 19.2–22.8°) and at 6 weeks was 16.40° (IQR 12.35–20.45°). The mean volar inclination before reduction was −14.18° ± 16.52°. After reduction, it was 5.00° (IQR 0.25–9.75°) and at 6 weeks was −2.50° (IQR −10.34–5.34°). The mean ulnar variance before reduction was 3.49 mm ± 2.41 mm; post-reduction, it was 1.70 mm (IQR 0.20–3.25 mm), and at 6 weeks it was 3.67 mm ± 2.74 mm. The change (∆) in radial inclination was 4.00° (IQR 0.97–7.02°); in volar inclination, it was 9.00° (IQR 2.70–15.45°); and in ulnar variance, it was 1.80 mm (IQR 0.50–3.10 mm). Detailed data are presented in Table 2.

### 3.2. Associations Between Instability Predictors and Outcomes

#### 3.2.1. Univariate Linear Regression Models

**Figure 2:**
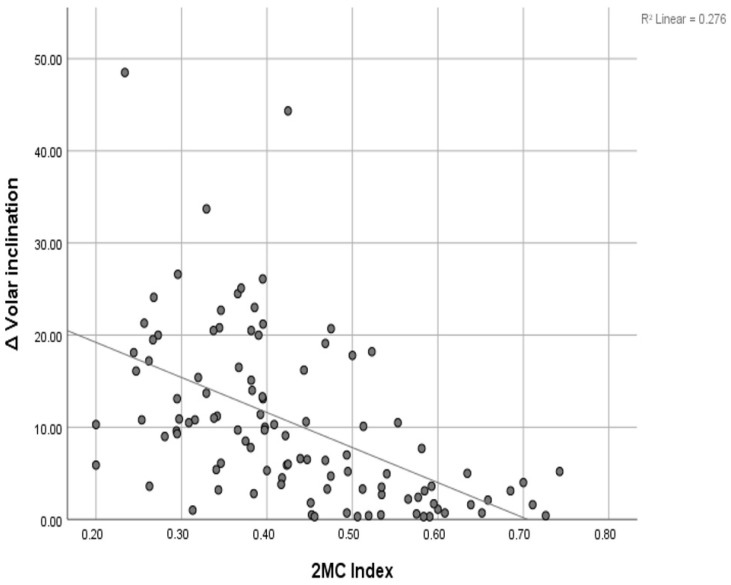
Linear regression ∆ volar inclination/2MCI.

Regarding ∆ volar inclination, univariate linear regression identified age (*p* = 0.002) and 2MCI (*p* < 0.001) as significant predictors. The linear relationship between ∆ volar inclination and 2MCI is depicted in Figure 2. Among categorical variables, the presence of an ulnar fracture (*p* = 0.045) and dorsal comminution (*p* = 0.003) were statistically significant.

Univariate analysis for ∆ radial inclination identified age (*p* < 0.001), 2MCI (*p* < 0.001), ulnar fracture (*p* = 0.025), and dorsal comminution (*p* = 0.047) as potential predictors.

∆ ulnar variation was significantly associated with age (*p* = 0.014, but with a low *R*^2^ of 5.8%), 2MCI (*p* < 0.001), ulnar fracture (*p* = 0.024), dorsal comminution (*p* < 0.001), and initial displacement (*p* = 0.007).

All statistical results from the univariate model can be found in Table 3.

#### 3.2.2. Multivariate Linear Regression Models

In the multivariate linear regression analysis for ∆ volar inclination, we included all the statistically significant covariates found in the univariate analysis (age, 2MCI, the presence of ulnar fracture, and dorsal comminution), as well as sex, considered a possible confounder. The overall model was statistically significant: *F* (5.99) = 8.12 with *p* < 0.001, explaining 29.1% of the variance in ∆ volar inclination (adjusted *R*^2^ = 25.5%). Among the included covariates, only 2MCI was independently associated with ∆ volar inclination (*B* = −42.533, β = −0.587 and *p* < 0.001), with lower 2MCI values predicting higher volar displacement at 6 weeks.

The multivariate analysis for ∆ radial inclination including the statistically significant covariates in the univariate regression (age, 2MCI, ulnar fracture, and dorsal comminution), as well as gender, was statistically significant (*p* < 0.001), explaining 25.5% of the variance (adjusted *R*^2^ = 21.7%). However, only 2MCI remained independently associated with ∆ radial inclination (*B* = −12.92, β = −0.366 and *p* = 0.004), indicating a moderate inverse relationship. Every unit increase in the 2MCI index predicts a 12.92° decrease in ∆ radial inclination.

The multivariate linear regression for ∆ ulnar variation identified an *R*^2^ = 25.2% (adjusted *R*^2^ = 20.6%) with statistical significance of the model (*p* < 0.001). This analysis identified 2MCI (*p* = 0.003), as well as initial displacement (*p* = 0.049), as an independent predictor for ∆ ulnar variation. Higher initial dorsal comminution was correlated with greater ulnar variance (B = 0.511, β = 0.138), whereas the relationship between the 2MCI and ∆ ulnar variation showed an inverse relationship (B = −5.208, β = −0.386). All the results of the multivariate regressions are presented in Table 4.

Among the three multivariate regression models, the one analyzing ∆ volar inclination demonstrated the highest explanatory power (*R*^2^ = 0.291 for ∆ volar inclination vs. 0.255 for ∆ radial inclination vs. 0.252 for ∆ ulnar variance). Considering the higher statistical power of the model, as well as the higher functional importance out of all the outcome variables, we decided to conduct an ROC analysis using ∆ volar inclination as the outcome and 2MCI as its predictor.

### 3.3. ROC Analysis

**Figure 3:**
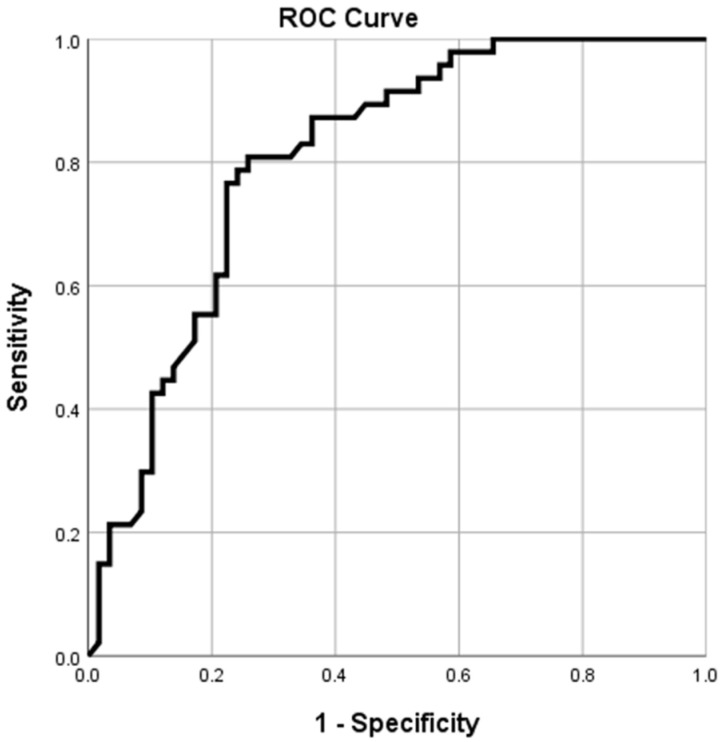
ROC analysis 2MCI predicting 10° ∆ volar inclination (AUC 80.6%, *p* < 0.001).

ROC analysis evaluating the MC2 index for predicting a ∆ volar inclination greater than 10° demonstrated a very good discriminative ability (*AUC* = 80.6% and *p* < 0.001). A 2MCI cut-off value of 0.412 optimized sensitivity at 80.9% and specificity at 74.1%, with a Youden J index of 0.55 (Figure 3).

## 4. Discussion

The epidemiology and the treatment of DRFs are strongly influenced by bone demineralization [1]. Bergh et al. reported that DRFs are the most common fractures in women over 50 years and the third most common in men over 50 years [18]. Shah et al. further noted that DRFs tend to occur earlier than vertebral fractures and up to 15 years earlier than hip fractures, placing them at the beginning of the fragility fracture sequence that can significantly affect patient quality of life and impose important healthcare and economic burdens [19]. Therefore, the occurrence of a DRF, particularly in people over 50 years of age, should be considered a good moment to initiate evaluation for osteoporosis.

The 2MCI is a simple and practical tool for screening osteopenia and osteoporosis, which can be easily used in emergency settings. Joseph et al. demonstrated that 2MCI strongly correlates with DEXA t-scores for the hip at a cut-off value below 60% (88% sensitivity and 60% specificity) to distinguish osteopenic from healthy bone, and a value less than 50% (100% sensitivity, 91% specificity) to differentiate osteoporotic patients from healthy ones [9]. More recently, in 2024, O’Mara et al. confirmed the utility of 2MCI as a predictor of osteoporosis, reporting that a cut-off value of 48.3% has 80% sensitivity and 79.2% sensibility for diagnosing osteoporosis of the distal third of the forearm [20].

Management of DRFs remains variable, with no universal consensus on the definition or on objective threshold values for the established radiographic or demographic criteria. Consequently, the decision between conservative or surgical treatment remains largely physician-dependent [5,21].

Our study aimed to investigate 2MCI and its threshold value as a predictor of secondary displacement and as an objective parameter to support decision making between treatment options. Our findings demonstrated a strong correlation between 2MCI and changes (∆) in volar inclination, radial inclination, and ulnar variance in univariate regression analysis. Moreover, in multivariate regression analysis, 2MCI was tested together with other statistically significant covariates and potential cofounders such as sex. The 2MCI was an independent predictor of ∆ volar inclination and ∆ radial inclination. For ∆ ulnar variance, both 2MCI and initial displacement were statistically significant predictors.

We conducted an ROC analysis on the strongest multivariate model (∆ delta volar inclination). The ROC analysis demonstrated a 2MCI cut-off value of 0.412, which predicted a ∆ volar inclination greater than 10°, a change considered clinically relevant for functional outcomes.

Our findings are in agreement with the results reported by Ulmer et al., who also identified 2MCI as an independent predictor for changes in volar inclination. Furthermore, they identified a 2MCI cut-off value of 53.5% as predictive of a fracture displacement in sagittal plane of 10°, while a cut-off value of 49.5% predicts 20° of dorsal tilt [22]. In addition, Ghodasra et al. have reported a statistically significant association between 2MCI with values in the osteoporotic range and an increase in ulnar variance [23].

In clinical practice, 2MCI can be easily and systematically assessed in the emergency department or outpatient clinic, providing valuable insights into the possible outcomes of a DRF. A value under the threshold value of 0.412 can be used to decide on surgical treatment, while above this value, conservative treatment should be considered. Consequently, it represents an important argument when deciding between conservative and surgical treatment. Improving decision-making protocols may ultimately reduce complications associated with both treatment options. For conservative treatment, correctly identifying patients at risk of fracture instability reduces the incidence of secondary displacement and the need for delayed surgical intervention or correction osteotomies. Meanwhile, for surgical treatment, more precise selection of patients can decrease surgical-related complications, particularly in borderline cases.

Given that published data on surgical vs. conservative treatment outcomes in elderly patients is sometimes contradictory, Chung et al., in the WRIST trial, reported that surgical-treated patients had better functional scores during the early follow-up period, but by 12 months, these differences had disappeared regardless of the treatment option. In contrast, Haslhofer et al., in a 2024 randomized trial, reported better functional outcomes for surgical treatment compared with non-surgical management [24,25]. Improving tools to stratify the risk of secondary displacement in DRFs might help optimize functional recovery in both treatment approaches.

Moreover, even in the absence of a DRF, the 2MCI predictive value for osteoporosis can be easily used by orthopedic surgeons or primary care practicians as a screening tool for early identification of patients at risk. This would allow timely referral for specialized evaluation and initiation of osteoporosis treatment, potentially preventing fragility fractures [26].

Given the promising results of this study, 2MCI shows potential as a practical tool to guide treatment selection in DRFs, as well as a screening tool for osteoporosis. Its systematic use could provide the opportunity for early detection and intervention in patents at risk of osteoporosis and fragility fractures. We believe that 2MCI could become a routinely assessed radiographic measurement whenever hand radiographs are obtained by an orthopedic surgeon, rheumatologist, general practitioner, or other clinician. However, to further strengthen its clinical applicability and establish its use as a standard decision-making tool, prospective randomized studies with larger cohorts and longer follow-up periods assessing both radiographic and functional outcome are warranted.

There are several limitations to our study. First, its retrospective design may introduce a selection bias, as only patients with available and complete radiographic follow-up were included and detailed data regarding the initial trauma mechanism and a complete list of comorbidities were not available for all included patients. Second, there was limited information on patient compliance with conservative treatment, specifically, adherence to immobilization protocols or instances where immobilization may have been removed intermittently or damaged. In a few such cases, where data was available, these patients were excluded from analysis. Furthermore, the short period of follow-up, while adequate to evaluate early secondary displacement, does not account for late remodeling or long-term functional outcomes. Finally, the current statistical model does not account for these potential confounding risk factors, which could influence the observed outcomes.

## 5. Conclusions

The results of this study demonstrate a strong correlation between the second metacarpal index and the instability of distal radius fractures. The 2MCI shows potential as a simple and objective tool, in addition to the Lafontaine criteria, in assessing fracture stability and guiding the choice between conservative and surgical management.

To our knowledge, there are limited studies evaluating the predictive value of 2MCI in DRF instability and its clinical importance. Therefore, before it can be adopted as a standard clinical tool, it would be of real value to validate it through a prospective randomized trial.

## Figures and Tables

**Table 1:** Demographic and fracture characteristics.

Variable	Category	n (%) = 105
**Age (years)**	Median (IQR)	68.00 (19.00)
	Range	20–90
**Sex**	Male	23 (21.9)
	Female	82 (78.1)
**AO Classification**	A1	2 (1.9)
	A2	21 (20)
	A3	58 (55.2)
	B1	6 (5.7)
	B2	1 (1.0)
	B3	4 (3.8)
	C1	6 (5.7)
	C2	3 (2.9)
	C3	4 (3.8)
**Intra-articular fracture**	Yes	24 (22.9)
**Dorsal comminution**	Yes	73 (69.5)
**Initial displacement (>20° dorsal tilt)**	Yes	39 (37.1)
**Associated ulnar fracture**	Base	37 (35.2)
	Neck	3 (2.9)
	Tip	13 (12.4)
	No fracture	52 (49.5)

**Table 2:** Descriptive statistics of radiographic parameters.

Parameter	Measurement Time	Mean ± SD	Median (IQR)
**2MCI**			0.40 (0.32–0.49)
**Radial Inclination (°)**	Pre-reduction	16.65 ± 6.31	
	Post-reduction		21.00 (19.2–22.8)
	6 weeks		16.40 (12.35–20.45)
**Volar Inclination (°)**	Pre-reduction	−14.18 ± 16.52	
	Post-reduction		5.00 (0.25–9.75)
	6 weeks		−2.50 (−10.34–5.34)
**Ulnar Variance (mm)**	Pre-reduction	3.49 ± 2.41	
	Post-reduction		1.70 (0.20–3.25)
	6 weeks	3.67 ± 2.74	
**∆ Radial Inclination (°)**			4.00 (0.97–7.02)
**∆ Volar Inclination (°)**			9.00 (2.70–15.45)
**∆ Ulnar variance (mm)**			1.80 (0.50–3.10)

**Table 3:** Univariate linear regression analyses for predictors of displacement at 6 weeks.

Dependent Variable	Predictor	R^2^	Adjusted R^2^	B	Standard Error	β	95% CI	t	*p*-Value
**∆Volar inclination**	Age	0.089	0.080	0.179	0.057	0.298	0.067–0.291	3.165	0.002
	2MCI	0.276	0.269	−38.049	6.076	−0.525	−50.100–−25.998	−6.262	<0.001
	Ulnar fracture	-	-						0.045
	Dorsal comminution	-	-						0.003
	Intra-articular fracture	-	-						0.11
	Initial displacement	-	-						0.16
	Sex	-	-						0.08
**∆ Radial inclination**	Age	0.147	0.139	0.113	0.027	0.384	0.060–0.165	4.216	<0.001
	2MCI	0.233	0.225	−17.042	3.050	−0.482	−23.090–−10.993	−5.587	<0.001
	Ulnar fracture	-	-						0.025
	Dorsal comminution	-	-						0.047
	Intra-articular fracture	-	-						0.68
	Initial displacement	-	-						0.86
	Sex	-	-						0.98
**∆ Ulnar Variation**	Age	0.058	0.049	0.027	0.011	0.240	0.006–0.048	2.512	0.014
	2MCI	0.162	0.154	−5.433	1.216	−0.403	−7.845–−3.022	−4.468	<0.001
	Ulnar fracture	-	-						0.024
	Dorsal comminution	-	-						<0.001
	Intra-articular fracture	-	-						0.33
	Initial displacement	-	-						0.007
	Sex	-	-						0.87

**Table 4:** Multivariate model linear regression analyses.

Outcome		R^2^	Adjusted R^2^	F	B	Standard Error	β	95% CI	t	*p*
**∆ Volar inclination**		0.291	0.255	8.129						<0.001
	Age				−0.083	0.072	−0.138	−0.226–0.060	−1.155	0.25
	2MCI				−42.533	8.829	−0.587	−60.052–−25.013	−4.817	<0.001
	Ulnar fracture				0.019	1.595	0.001	−3.146–3.184	−0.012	0.99
	Dorsal comminution				1.629	1.778	0.082	−1.900–5.157	0.916	0.36
	Sex				1.079	2.016	0.049	−2.920–5.079	0.593	0.59
**∆ Radial inclination**		0.255	0.217	6.772						<0.001
	Age				0.024	0.036	0.081	−0.048–0.095	0.662	0.51
	2MCI				−12.923	4.414	−0.366	−21.682–−4.165	−2.928	0.004
	Ulnar fracture				0.621	0.797	0.069	−0.962–2.203	0.778	0.43
	Dorsal comminution				0.932	0.889	0.096	−0.832–2.696	1.048	0.29
	Sex				0.929	1.008	0.086	−1.071–2.928	0.359	0.35
**∆ Ulnar variance**		0.252	0.206	5.494						<0.001
	Age				−0.003	0.014	−0.031	−0.031–0.024	−0.248	0.80
	2MCI				−5.208	1.697	−0.386	−8.575–−1.841	−3.069	0.003
	Ulnar fracture				0.360	0.308	0.106	−0.252– 0.973	1.168	0.24
	Dorsal comminution				0.511	0.349	0.138	−0.182–1.203	1.462	0.14
	Initial displacement				0.637	0.319	0.180	0.003–1.271	1.995	0.049
	Sex				−0.251	0.387	−0.061	−1.019–0.518	−0.648	0.51

## Data Availability

The data is available upon request from the corresponding author.

## References

[1] Nellans K.W., Kowalski E., Chung K.C. (2012). The Epidemiology of Distal Radius Fractures. Hand Clin..

[2] Diaz-Garcia R.J., Chung K.C. (2012). The Evolution of Distal Radius Fracture Management—A Historical Treatise. Hand Clin..

[3] Alanazi A.A., Alsharari A.M., Alrumaih N.H., Alsudays A.I., Alanazi A.K., Alhilali M., Bo Shagea F., Al-Rawaf M.M., Alsiwat F.J. (2024). Surgical vs. Conservative Treatment of Distal Radius Fractures in the Elderly: A Systematic Review and Meta-Analysis. Cureus.

[4] Diepold J., Filipp S., Dussing F., Steiner G., Deininger C., Gotterbarm T., Wichlas F. (2025). Poor Fracture Alignment Equals Poor Outcome? Analysis of Conservatively Managed Distal Radius Fractures. Arch. Orthop. Trauma Surg..

[5] Walenkamp M.M.J., Vos L.M., Strackee S.D., Goslings J.C., Schep N.W.L. (2015). The Unstable Distal Radius Fracture—How Do We Define It? A Systematic Review. J. Wrist Surg..

[6] Salari N., Darvishi N., Bartina Y., Larti M., Kiaei A., Hemmati M., Shohaimi S., Mohammadi M. (2021). Global Prevalence of Osteoporosis among the World Older Adults: A Comprehensive Systematic Review and Meta-Analysis. J. Orthop. Surg. Res..

[7] Mantila Roosa S.M., Hurd A.L., Xu H., Fuchs R.K., Warden S.J. (2012). Age-Related Changes in Proximal Humerus Bone Health in Healthy, White Males. Osteoporos. Int..

[8] Gullborg E.J., Kim J.H., Ward C.M., Simcock X.C. (2024). Optimizing Treatment Strategies for Distal Radius Fractures in Osteoporosis: A Comparative Review. Medicina.

[9] Schreiber J.J., Kamal R.N., Yao J. (2017). Simple Assessment of Global Bone Density and Osteoporosis Screening Using Standard Radiographs of the Hand. J. Hand Surg..

[10] Kitidumrongsook P., Luangjarmekorn P., Kuptniratsaikul V., Teeragananan T., Chaitantipongse S. (2024). Measurement of Radiological Parameters of Distal Radius Fracture Using the Ulnar Axis Compared with the Radial Axis. J. Hand Surg. (Asian-Pac. Vol.).

[11] Pace V., Lanzetti R.M., Venditto T., Park C., Kim W.J., Rinonapoli G., Caraffa A. (2021). Dorsally Displaced Distal Radius Fractures: Introduction of Pacetti’s Line as Radiological Measurement to Predict Dorsal Fracture Displacement. Acta Biomed..

[12] Lichtman D.M., Bindra R.R., Boyer M.I., Putnam M.D., Ring D., Slutsky D.J., Taras J.S., Watters W.C., Goldberg M.J., Keith M. (2011). American Academy of Orthopaedic Surgeons Clinical Practice Guideline on: The Treatment of Distal Radius Fractures. J. Bone Jt. Surg..

[13] Bong M.R., Egol K.A., Leibman M., Koval K.J. (2006). A Comparison of Immediate Postreduction Splinting Constructs for Controlling Initial Displacement of Fractures of the Distal Radius: A Prospective Randomized Study of Long-Arm Versus Short-Arm Splinting. J. Hand Surg..

[14] Farah N., Nassar L., Farah Z., Schuind F. (2014). Secondary Displacement of Distal Radius Fractures Treated by Bridging External Fixation. J. Hand Surg. (Eur. Vol.).

[15] Elbardesy H., Yousaf M.I., Reidy D., Ansari M.I., Harty J. (2023). Distal Radial Fractures in Adults: 4 versus 6 Weeks of Cast Immobilisation after Closed Reduction, a Randomised Controlled Trial. Eur. J. Orthop. Surg. Traumatol..

[16] Okamura A., de Moraes V.Y., Neto J.R., Tamaoki M.J., Faloppa F., Belloti J.C. (2021). No Benefit for Elbow Blocking on Conservative Treatment of Distal Radius Fractures: A 6-Month Randomized Controlled Trial. PLoS ONE.

[17] Meinberg E.G., Agel J., Roberts C.S., Karam M.D., Kellam J.F. (2018). Fracture and Dislocation Classification Compendium-2018. J. Orthop. Trauma.

[18] Bergh C., Wennergren D., Möller M., Brisby H. (2020). Fracture Incidence in Adults in Relation to Age and Gender: A Study of 27,169 Fractures in the Swedish Fracture Register in a Well-Defined Catchment Area. PLoS ONE.

[19] Shah G.M., Gong H.S., Chae Y.J., Kim Y.S., Kim J., Baek G.H. (2020). Evaluation and Management of Osteoporosis and Sarcopenia in Patients with Distal Radius Fractures. Clin. Orthop. Surg..

[20] O’Mara A., Kerkhof F., Kenney D., Segovia N., Asbell P., Ladd A.L. (2024). Opportunistic Hand Radiographs to Screen for Low Forearm Bone Mineral Density: A Prospective and Retrospective Cohort Study. BMC Musculoskelet. Disord..

[21] Lafontaine M., Hardy D., Delince P. (1989). Stability Assessment of Distal Radius Fractures. Injury.

[22] Ulmer C.J., Verlinsky L., Emukah C.C., Ogburn M.J., Ubanwa B., Sager B.W. (2025). Rethinking Lafontaine Criteria: Second Metacarpal Cortical Percentage as a Reliable Predictor of Distal Radius Fracture Instability. Hand.

[23] Ghodasra J.H., Yousaf I.S., Sanghavi K.K., Rozental T.D., Means K.R., Giladi A.M. (2021). Assessing the Relationship Between Bone Density and Loss of Reduction in Nonsurgical Distal Radius Fracture Treatment. J. Hand Surg..

[24] Chung K.C., Kim H.M., Malay S., Shauver M.J. (2020). The Wrist and Radius Injury Surgical Trial (WRIST): 12-Month Outcomes from a Multicenter International Randomized Clinical Trial. Plast. Reconstr. Surg..

[25] Haslhofer D.J., Froschauer S.M., Gotterbarm T., Schmidt M., Kwasny O., Holzbauer M. (2024). Comparison of Surgical and Conservative Therapy in Older Patients with Distal Radius Fracture: A Prospective Randomized Clinic al Trial. J. Orthop. Traumatol..

[26] Ong J., Snee I., Marcano I., Tintle S., Cheikh M., Giladi A.M. (2025). Bone Health, Fragility Fractures, and the Hand Surgeon. J. Hand Surg. Glob. Online.

